# Identification of evolutionarily stable sites across the SARS-CoV-2 proteome

**DOI:** 10.21203/rs.3.rs-95030/v1

**Published:** 2020-10-20

**Authors:** Chen Wang, Daniel M. Konecki, David C. Marciano, Harikumar Govindarajan, Amanda M. Williams, Brigitta Wastuwidyaningtyas, Thomas Bourquard, Panagiotis Katsonis, Olivier Lichtarge

**Affiliations:** Baylor College of Medicine; Baylor College of Medicine; Baylor College of Medicine; Baylor College of Medicine; Baylor College of Medicine; Baylor College of Medicine; Baylor College of Medicine; Baylor College of Medicine; Baylor College of Medicine

**Keywords:** SARS-CoV-2, COVID-19, coronavirus, epitopes, sequence analysis

## Abstract

Since the first recognized case of COVID-19, more than 30 million people have been infected worldwide. Despite global efforts in drug and vaccine development to fight the disease, there is currently no vaccine or drug cure for COVID-19, though some drugs reduce severity and hasten recovery. Here we interrogate the evolutionary history of the entire SARS-CoV-2 proteome to identify functional sites that can inform the search for treatments. Combining this information with the mutations observed in the current COVID-19 outbreak, we systematically and comprehensively define evolutionarily stable sites that are useful drug targets. Several experimentally-validated effective drugs interact with these proposed target sites. In addition, the same evolutionary information can prioritize cross reactive antigens that are useful in directing multi-epitope vaccine strategies to illicit broadly neutralizing immune responses to the betacoronavirus family. Although the results are focused on SARS-CoV-2, these approaches are based upon evolutionary principles and are agnostic to organism or infective agent.

## Introduction

COVID-19 is a worldwide affliction. Since first being reported in December 2019 in Wuhan, Hubei province, China, the World Health Organization (WHO) has tallied more than 950,000 COVID-19 related deaths and over 30 million infections worldwide (as of September 19^th^, 2020) (1). Although timely public health interventions can successfully curtail incidence, the threat of subsequent waves of infections remains widespread (1–3). The novel betacoronavirus (SARS-CoV-2) that is causing the pandemic is closely related to other known human coronavirus pathogens SARS-CoV, MERS-CoV (4, 5), HCoV OC43, HKU1 and is more distantly related to the human infectious alphacoronaviruses HCoV 229E and HCoV NL63 (6). Finding ways to control and prevent further infection are top priorities which include the targeted discovery of drugs that impair viral mechanisms (7–9) and antigenic epitopes through which vaccines raise immunity (10–12). This study addresses both by utilizing evolutionary information from SARS-CoV-2 sequence and structural data to search for actionable functional sites for each protein in the SARS-CoV-2 genome.

In a first application, we note that the approval of new drugs under normal circumstances often takes more than 10 years (13, 14). In order to hasten the response, many current clinical trials for COVID-19 enlist antiviral agents that have targeted Zika, SARS-CoV, Ebola, and MERS-CoV in the past (13, 15). In order to test more varieties of potential drugs, some studies screened thousands of clinical-stage or FDA-approved small molecules for antiviral activity, hoping to repurpose some of the top hits for COVID-19 treatment (16). However, the antiviral activity in these large-scale screens may, in part, be cell-line specific (17), and therefore of unclear clinical relevance. Another approach to screen potential drugs for repurposing is to perform docking (18) of clinical-stage or FDA-approved drugs to the SARS-CoV-2 proteome (19, 20). However, selection of the correct binding sites on the target proteins is crucial and difflcult as protein surface cavities far exceed actual ligand binding sites that modulate function (21). Here we systematically suggest potential drug target sites for most SARS-CoV-2 proteins based on evolutionary information. As these sites are chosen for their conserved functional roles, broad pan-coronavirus/betacoronavirus relevance, and minimal variability across all known current SARS-CoV-2 variants, they should be prioritized in docking studies for drug repurposing.

In a second application, we note that understanding the immune response to SARS-CoV-2 infection is critical for vaccine development (22). Most early SARS-CoV-2 immune epitope discovery studies rely heavily on bioinformatic prediction tools as well as sequence and epitope work already done in SARS-CoV and MERS-CoV. B-cell linear and discontinuous epitope prediction tools have been used by researchers to identify possible SARS-CoV-2 epitopes (23–25). Several more recent studies experimentally determined SARS-CoV-2 immune epitopes (11, 26, 27). Interestingly, several groups have reported significant T-cell reactivity against SARS-CoV-2 epitopes in individuals without virus exposure (22, 26, 28, 29). Mateus et al. suggested that this could be due to cross reactivity between SARS-CoV-2 and other common human coronaviruses, such as OC43, HKU1, NL63 and 229E (28). Here we report an evolutionary metric, which can accurately separate cross-reactive epitopes from those that are not, and use this metric to suggest potential cross-reactive epitopes in SARS-CoV-2. Prioritizing these cross-reactive epitopes in vaccine development can potentially lead to broadly neutralizing immunity across the betacoronavirus family.

Here, we use the Evolutionary Trace (ET) method, which predicts the importance of protein sequence positions, from most important (0.0) to least important (100.0). This relative ranking reflects the variation entropy of each sequence position within and across the branches of an associated phylogenetic tree, revealing evolutionary pressure points that correspond to functional and structural determinants, and the protein sites at which they often cluster (30). Past studies have shown that this method can predict binding and catalytic functional sites (31, 32), guide protein engineering (33, 34) and predict function (35). ET rankings of residue importance can also be combined with amino acid substitution log odds to estimate the likely impact, or Evolutionary Action (EA), of coding variations on protein function (36–38). Here, this first ET and EA analysis of a full viral proteome identifies evolutionary important residues and functional sites in the SARS-CoV-2 proteome.

## Results

### Evolutionary Trace of SARS-CoV-2.

In order to map functional determinants in SARS-CoV-2 proteins we applied the ET approach. With the multiple sequence alignments (Figure S1A, Dataset S1) and the corresponding phylogenetic trees (Figure S2–S4) in hand for 24 of the 26 SARS-CoV-2 proteins (see SI Methods and Materials), our protocol calculated the ET ranking of importance for 99.5% of SARS-CoV-2 amino acid residue positions (Dataset S2) generated from each of three protein databases (UniRef90, UniRef100, NCBI NR) and combined into a single average. To independently assess the quality of these ranks, rather than rely on the variety and breadth of sequences in the alignments as indicative of information content, we used a statistical measure that quantifies the distribution of ET rankings in the 3D structure. Residues with smaller ET rankings tend to cluster together in active sites, protein-protein interaction sites or other functional sites (30, 31, 39–41). Such a clustering of top-ranked residues was particularly prominent in several SARS-CoV-2 proteins and complexes including the NSP5 main protease, the NSP7/NSP8/NSP12 RNA-dependent RNA polymerase complex and the NSP10/NSP16 RNA cap methyltransferase complex and can be visualized as groups of warm colored residues in the protein structure (**Figure 1**). We evaluated the quality of ET rankings using the Selection Cluster Weighting (SCW) z-score which measures how well highly ranked residues cluster relative to a randomized distribution of scores on the structure (see SI Materials and Methods). For almost all proteins the SCW z-Score is 2 standard deviations above the randomized background, suggesting that the alignments are informative and that the resulting ET rankings are meaningful (Figure S1, Dataset S3). For the proteins that do not reach significant z-scores there is a clear correlation to a lack of sequences in the alignments (e.g. NSP1, E, ORF3, and ORF7a), or, the structure belongs to a small domain within a larger protein (e.g. the macrodomain within NSP3 and the HR2 domain within the S protein).

To probe these smaller domains within large proteins we further investigated the ADP-ribose-phosphatase (ADPRP) subdomain and macro and papain-like protease (PL^pro^) domains of NSP3. NSP3 was an intriguing case because top-ranked ET residues cluster well in its PL^pro^ domain but not in its macrodomain or in the ADPRP subdomain (Dataset S3). In order to better resolve ET rankings for NSP3, we generated new alignments, phylogenetic trees, and ET residue rankings for the subsequences specific to each NSP3 domain structure (see SI Materials and Methods). In this focused analysis, the PL^pro^ domain now yielded ~50% more sequences leading to a corresponding increase in the clustering of top-ranked residues (Figure S5). For the macrodomain and ADPRP subdomain, thousands of additional sequences spanning the three domains of life and distantly related viruses were included in the new data set which resulted in ET rankings that rivaled the significance of clustering in the PL^pro^ domain. The stark differences we find in the phylogenetic trees of specific NSP3 domains confirm previous observations of alternate domain configurations in different coronavirus genera and even within clades of betacoronavirus (6). The improvement in SCW z-score corresponds to a cluster of highly ranked ET residues within the ligand binding site of the macro domain and ADPRP subdomain (Figure S5D and E) which was missing in the analysis of the full NSP3 reference sequence. Having better resolved ET rankings in the NSP3 domains, we returned to the main data set to see how well ET rankings captured functional sites in other proteins.

### Phylogenetically conserved ligand binding sites.

A catalog of SARS-CoV-2 ligand binding sites could serve as a timely resource for prioritizing therapeutic targets. Previous studies have shown that evolutionary sequence information correlates well-enough with enzyme active sites so as to serve as 3D-templates for functional signatures (35) and identify allosteric sites (42, 43). Here we used NSP12, NSP15 and NSP16 as examples to show how the evolutionary sequence information captured by ET can successfully predict ligand binding sites for virus proteins. NSP12 is an RNA dependent polymerase, NSP15 mediates the cleavage of both single- and double-stranded RNA at uridine sites (44) and NSP16 is a m7GpppA-specific, S-adenosylmethionine (SAM)-dependent, 2’-O-MTase (45). As shown in **Figure 2A-C**, top ranked ET residues cluster around the native ligands of NSP12 (RNA) (46), NSP15 (GpU) (8) and NSP16 (m7GpppA and SAM) (47), indicating an accurate prediction of ligand binding sites for these proteins. Several new functional sites are also predicted by ET (**Figure 2D and 2E**). On the spike protein (S), one such ET cluster partially overlaps the S2’ protease cleavage site that is critical for membrane fusion and infectivity of the SARS virus (48). On the nucleoprotein (N), a cluster of highly ranked ET residues lies adjacent to the putative RNA binding site (49) and may contribute to formation of N protein-RNA helical laments that are essential to packaging the RNA genome. These results indicate ET can provide alternative drug target sites with no currently available ligand-bound structures.

In addition to being important to protein function, ideal drug target sites should also be rarely mutated in the current outbreak so as to avoid the potential emergence of drug resistance. Thus, we focused on positions that do not have any mutations observed in the 52,061 high quality, full length SARS-CoV-2 sequences that were available as of September 14th, 2020. As more genomes and mutations within them are sequenced it may be necessary to lower the variant count stringency. In order to translate proteome-wide ET ranks and mutational pro les into potential drug target sites, we focused on clusters of mutationfree, surface-exposed residues that are highly ranked by ET and fall within 5Å of each other (**Figure 3,** Dataset S4). The resulting catalog of putative drug targets includes 116 sites at ~5 sites per structure with the largest structure (full-length model of Spike, 6vsb_1_1_1) having the highest number of sites. For NSP12, NSP15 and NSP16, the predicted drug targets overlap the known ligand binding sites.

In order to evaluate whether these ET drug sites may correspond to druggable target sites, we examined their overlap with sites observed in five SARS-CoV-2 protein-drug complex crystal structures. It is important to note that all 5 drugs showed an inhibitory effect in either cellular or biochemical assays. Remdesivir has been shown to speed up the recovery of COVID-19 patients in clinical trials (50), while the α-ketoamide inhibitor 13b can suppress SARS-CoV-2 replication in cell lines (51). Vir251 and tipiracil were also shown to effectively inhibit the enzymatic activities of their targets (7, 8). The remaining drug, sinefungin, is a pan-MTnase (NSP16) inhibitor that inhibits the growth of yeast cells ectopically expressing NSP16 from SARS-CoV (45). The ET drug sites were mapped onto the five SARS-CoV-2 protein-drug complexes (7, 8, 51–53) and, as shown in **Figure 3**, all five drugs reside in protein surface pockets that are within or very close to our predicted ET drug sites. The ET drug site for NSP5 is the least well recovered due to a single SARS-CoV-2 sequencing entry (strain MT745875) wherein several residues in the protease active site are mutated (G143S, S144E and C145I), including the catalytic cystine residue. S144E and C145I are both caused by two nucleotide substitutions in the codon, and only observed in this strain (sampled on 06/24/20). It is unclear whether this is a sequencing artifact or represents a genuine active site plasticity that compromises NSP5’s active site as a stable drug target. It does however illustrate the importance of accurately detecting emerging sequence variations when choosing drug targets. Overall, these results show that predicted ET drug sites can recover experimentally tested drug binding pockets and suggest new sites that can be targeted in computational docking approaches. In addition, because these sites are conserved across multiple coronavirus genera, these predicted ET drug sites are anticipated to be relevant for identifying inhibitors of SARS-CoV-2 as well as more distantly related coronaviruses.

### Conserved linear epitopes.

ET drugs sites may prove valuable in guiding drug design, but these approaches are dependent upon having high resolution crystal structures and some structures are either not yet available (e.g. NSP2, NSP6, M, and several accessory proteins), do not cover a majority of the protein (NSP3 and NSP4) or are too low in resolution for accurate docking studies (NSP12, NSP14, ectodomain of S, N, ORF3a and ORF7a). However, ET operates over linear protein sequences and thereby can identify phylogenetically important sequence fragments even in the absence of a 3D structure (54). As in our approach to discover ET drug sites, we combined ET residue ranking information with sequencing data from SARS-CoV-2 isolates to arrive at linear peptides along the proteome that are evolutionarily important and also show little variation in the current outbreak (Figure S6, Dataset S5). In order to assess the value of these epitopes, we asked whether they could recapitulate ET-derived drug sites. ET-defined linear peptides for NSP12 were mapped onto an available NSP12 structure and, as illustrated in **Figure 4A**, the majority of the structural and linear peptides overlap with each other. Linear ET peptides and ET drug sites overlap well for other SARS-CoV-2 proteins, which was quantified by Jaccard Similarity and Fisher’s exact test (Dataset S6). These data suggest that linear ET peptides contain functionally relevant information since they recapitulate ET drug sites for proteins or domains without requiring 3D structural data. In the absence of a protein structure, these ET peptides could be useful in designing inhibitory peptides (55, 56).

These peptides are also connected to a second main approach towards resolving the pandemic, by way of vaccine development. Although vaccines for COVID-19 may become available soon, ideally, effective protection against future outbreaks from related coronaviruses would require a broadly neutralizing effect wherein the immune system recognizes epitopes shared among coronavirus species. The prospect of raising a broadly neutralizing response is bolstered by a recent study wherein naïve patients, never exposed SARS-CoV-2, were found to possess a subset of T-cells that can cross-react to homologous epitopes shared by common cold coronaviruses and SARS-CoV-2 (28). In this context, we note that ET rankings reflect the degree of homology over the phylogenetic tree, so we reasoned that summing ET scores over the length of an identified T-cell epitope may be able to estimate its potential for cross-reactivity.

As a first step, we summed the ET ranks for each of the 40 SARS-CoV-2 epitopes that had been shown to react with patient-derived T-cells so that they could be ranked by predicted cross-reactivity to 161 common cold coronavirus epitopes assayed by Mateus et al. Although summing ET ranks could identify SARS-CoV-2 epitopes that are more likely to be cross-reactive (Figure S7), it did not account for the specific amino acid differences in the potentially cross-reactive homolog. In other words, ET ranks can predict whether or not a SARS-CoV-2 epitope will be cross-reactive in general, but they do not specify which epitope homologs will cross react.

In order to improve resolution of our predictions to specific epitope homologs, we next combined EA, a predictor of mutational impact, with the summed ET rankings. EA calculates the predicted impact of amino acid variations on protein function aiding in the interpretation of coding variants (36–38). Summing the predicted impact of amino acid changes between a SARS-CoV-2 epitope and a homologous epitope in another virus (sumEA) while adjusting for the SARS-CoV-2 epitope’s overall evolutionary importance (sum(100-ET ranking)) produced a metric that was able to separate cross-reactive epitopes from those that did not cross react (**Figure 4B and** S7, Dataset S7). This metric, sumEA/sum(100-ET ranking), was then applied to 21 untested SARS-CoV-2 T-cell epitopes and their common cold homologs (28). From a total of 92 homologs we identified 23 with potential to cross react to one of five SARS-CoV-2 epitopes (**Figure 4C,** Dataset S8). These 5 SARS-CoV-2 epitopes along with the 9 others experimentally shown to possess cross-reactivity could be used in a multi-epitope vaccination strategy that provides a broad neutralizing response to currently circulating coronaviruses, SARS-CoV-2 and, possibly, future outbreaks. Moreover, the approach is not specifically linked to any specific virus, so it could be replicated in other families of pathogens.

### Dissemination.

To disseminate these results, a public website (http://cov.lichtargelab.org) makes these data and analyses fully accessible. The data include, for example, multiple sequence alignments, precalculated ET ranks, and predicted epitopes (both linear and structural) for all SARS-CoV-2 proteins. In addition, an interactive structure viewer enables users to explore any one of the ET-colored structures (**Figure 1**) and predicted ET drug sites associated with those structures (Dataset S4–5). The website will be updated as new SARS-CoV-2 isolates and protein structures become available.

## Discussion

Rapid progress has been made in response to the acute SARS-CoV-2 threat; from sequencing, to structural determination, and to drug and vaccine development (9, 57–60). Here, by combining information from evolutionary history and the current outbreak of SARS-CoV-2 we systematically mapped potential therapeutic sites on all SARS-CoV-2 proteins. We make use of phylogenetics, sequence information and structure information to provide a functional map of SARS-CoV-2 proteins. The sites we determined are not only stable across coronavirus families but are also stable to mutations in the current pandemic, which make them ideal targets for pan coronavirus/betacoronavirus therapeutics. In so doing, we pinpoint functionally and structurally important sites in the SARS-CoV-2 proteome that reduce the search space for drug and vaccine development. In addition to focusing therapeutic studies, the data presented here will be important in identifying the mechanism of action for successful therapies, not only in the context of the current outbreak but across future coronavirus outbreaks. Our findings are available on the accompanying website, where results will be updated as more SARS-CoV-2 isolates are sequenced, and structures are completed. This should not only expand coverage of the SARS-CoV-2 proteome and refine predicted therapeutic sites, but also provide a resource to monitor for variants that may significantly impact the virulence of SARS-CoV-2.

There are limitations to this study. The quality of our results depends on the number and range of homologous sequences available. Although most of the non-structural proteins yield ET rankings that are likely informative (clustering z-score >=2 or >30 unique sequences between 25–98% identity), NSP1 and the accessory proteins do not reach significant z-scores or have many diverse sequences in their final alignments. The inability to recover more sequence information could be due to a higher evolutionary rate in these proteins that limits our ability to recognize distantly related homologs with very little sequence identity. More likely, these peripheral genes have been more recently recruited through the frequent recombination events that occur in the coronavirus family (61). Such recruitment has occurred at the domain level in the NSP3 protein with its variable number of domains (10 to 16), some of which are unique to the betacoronavirus clade b containing SARS-CoV-1 and −2. Therefore, it is unsurprising that the initial sequences returned and corresponding ET rankings for full-length NSP3 are heavily influenced by the less divergent PL^pro^ domain that is present across coronavirus clades and families. Domain-specific analysis of NSP3 greatly improved both the number of sequences returned, phylogenetic coverage, and the resolution of ET results. This suggests that future work should include domain specific analyses for multidomain proteins. Such domain specific analyses are likely to provide ET rankings that identify important functional sites for individual domains while full-length analysis can provide insight into how particular domains became recruited for specific branches of the phylogenetic tree.

Several other groups have focused on experimentally screening clinical-stage or FDA-approved small molecules with the hope of identifying and repurposing drugs for SARS-CoV-2 treatment. Tens to hundreds of drug candidates are identified by these high-throughput assays. However, drug efficacy of top hits might be cell line specific (17) and the mechanisms of drug action may be unclear or acting through modulation of the host cell rather than targeting the virus itself. In silico docking studies (19, 62) take a more targeted approach towards specific SARS-CoV-2 sites that may complement the results of experimental screens. Knowledge of the ligand binding site improves the chance of identifying drugs that inhibit protein function and although structural characterization of SARS-CoV-2 proteins is unprecedented, the structural information available is far from comprehensive. Using the structures which have been solved, we identified clusters of surface residues that have low ET rankings and a lack of mutations in the current outbreak as potential drug target sites. Many of these ET drug sites correspond to ligand bound active sites but others map to evolutionarily important sites that have yet to be fully characterized. ET operates over the phylogenetic history of linear sequence space and can anticipate functional sites that may or may not be characterized in the future. These putative ET drug targets can guide docking studies to additional sites not immediately apparent from currently available structural information.

Sites highlighted by ET are evolutionarily conserved in the phylogenetic tree used in ET calculation and this information can set expectations for how broadly a drug may inhibit different viral species. For instance, Remdesivir targets the active site of RNA-dependent RNA polymerase (NSP12) in SARS-CoV-2 as well as homologs in SARS, MERS and the distantly related Ebola RNA virus (63, 64). The NSP12 active site has a very strong ET signal that is derived from one of deepest phylogenetic trees in our analysis and thereby would be expected to inhibit a wide swath of coronaviruses and related RNA viruses. In contrast, the ADP ribose phosphatase sub-domain of NSP3 has a phylogenetic tree that includes relatively few coronavirus sequences among a multitude of sequences that span three domains of life. Drugs targeting this domain may inhibit coronavirus infectivity but could also have side effects if they inhibit host ADP ribose phosphatases. However, ADP ribose phosphatase inhibitors have been developed for cancer treatment and a wealth of information and expertise is available for this group of drugs (65). As with the application of any new drug, particular care should be taken to ensure unwanted side effects do not overshadow any benefits as a viral inhibitor.

The linear epitopes we defined here may also provide valuable information in drug development both for proteins with structure, and for those without, as amino acids connected linearly are guaranteed to be connected structurally. For protein regions that are flexible or undergo large conformational changes during activation, structural proximity defined in one conformation may not hold in other conformations. For example, the Spike protein undergoes a large conformational change when mediating host-virus membrane fusion (66). A structural epitope that is determined in the closed state might not be appropriate for the opened state. Thus, linearly connected regions may identify cryptic binding sites that are revealed upon conformational change of the protein.

Linear epitopes are also a predominant mode of recognition of the adaptive immune system. Studies have shown that some SARS-CoV-2 T-cell epitopes are capable of cross reacting with homologous peptides in other human coronaviruses (26, 28). We performed evolutionary analysis on these cross-reactive epitopes and developed a new metric that can distinguish cross reactive epitopes with a high accuracy that outperforms a simple percent identity metric. This sumEA/sum(100-ET ranking) metric was then used to suggest other potential SARS-CoV-2 cross-reactive T-cell epitopes. In general, cross-reactive epitopes have the potential of generating a pan-betacoronavirus immune response that can stimulate B-cells to produce broadly neutralizing antibodies. Although not directly addressed in this work, the sumEA/sum(100-ET ranking) metric may also be able to identify epitopes that stimulate cytotoxic T-cells through presentation on MHC-1 molecules. Several groups are at the preclinical stage in multi-epitope vaccine development (milkeninstitute.org) but the specific epitopes are not publicly available, and it is unknown whether or not they include any that are cross reactive. The ability to identify cross-reactive epitopes could inform a multi-epitope vaccine strategy that is specifically designed to inoculate a susceptible population to a wide range of extant and undiscovered betacoronaviruses.

## Conclusion

This study was motivated by the current pandemic and uses evolutionary sequence information to guide the development of therapeutics for COVID-19. Although we are presently in the grip of COVID-19, this pandemic was preceded by the SARS and MERS outbreaks and it should be anticipated that related coronaviruses will cause future outbreaks. And while this study is also focused upon SARS-CoV-2, it draws upon pieces of sequence information taken from the whole of the coronavirus family and thereby the ndings are extendable to other coronavirus species, including those that have not yet been encountered. Indeed, the tools we present could be applied to any family of pathogen. Putting a pandemic virus into the evolutionary context of related viruses can expose a path to managing a recovery and may offer therapeutics that cover future outbreaks.

## Materials And Methods

A brief description of the methods can be found here, for a more in-depth description of specific methods please see the Supplementary text.

### Evolutionary Trace:

In order to map functional determinants in SARS-CoV-2 proteins we applied the Evolutionary Trace (ET) approach (30, 31). This method ranks each amino acid position from most to least important during evolution by tracking how they vary along the coronavirus phylogenetic tree. These rankings vary based on the precise choice of multiple sequence alignment (MSA). In order to produce robust ET rankings three separate alignments were generated for each protein in the SARS-CoV-2 Wuhan-Hu-1 reference genome (NC_045512.2) (57), by querying three protein databases (UniRef90, UniRef100, and NCBI NR) for sequences with identity between 25% and 98%, thus ltering out those that were either overly distant or redundant. Only two proteins had too few matches for ET, NSP11 and ORF10, both of which have unknown function and have very short reference sequences (13 and 38 amino acids, respectively, FigureS1, Dataset S1). The ET scores for all other proteins for each alignment and for the average scores across alignments were evaluated with the previously presented Selection Cluster Weighting (SCW) *z*-score (30, 39–41). The z-scores for each structure were then ranked 1–4 in order to determine if ET scores from one database or the average of the three consistently outperforms the others. ET scores from each of the three databases performed similarly well but the average ET of the three provided better z-scores in most cases (Figure S1C). ET rankings were further investigated by comparing the highest scoring regions with known functional sites.

### Prediction of Therapeutic Sites:

Therapeutic sites were predicted based on both the linear sequence as well as structural constraints. Residues were nominated as members of potential therapeutic sites based on their ET rankings, lack of variants as found in SARS-CoV-2 sequences retrieved from GISAID (67) and the China National Center for Bioinformation (68)(CNCB), as well as surface accessibility, and structural proximity. Structurally identified therapeutic sites were compared to drug binding sites for agents known to bind to SARS-CoV-2 proteins. To generalize this approach to proteins without structure, linear sites were predicted based on ET rankings, current mutational profile and linear connectivity. Structural and linear predicted sites were compared to one another using Jaccard Similarity and Fisher’s Exact test, to determine the usefulness of this method in the absence of a protein structure. Several ET metrics were also interrogated to determine their ability to highlight potential cross-reactive immunogenic epitopes (28). The best metric, sumEA/sum(100-ET ranking), was used to predict cross-reactive T-cell epitopes which are good potential therapeutic sites.

## Supplementary Material

Supplement

Supplement

Supplement

Supplement

Supplement

Supplement

Supplement

Supplement

Supplement

## Significance Statement

By examining past evolutionary pressures in the coronavirus family and in the present SARS-CoV-2 outbreak, we identified functional sites in the SARS-CoV-2 proteome that can be targeted for small molecule docking, used for pan-coronavirus/betacoronavirus vaccine development, provide templates for mimetic peptides, and offer genetic targets to generate attenuated virus.
