# Identification of evolutionarily stable functional and immunogenic sites across the SARS-CoV-2 proteome and the greater coronavirus family

**DOI:** 10.21203/rs.3.rs-95030/v3

**Published:** 2021-02-15

**Authors:** Chen Wang, Daniel M. Konecki, David C. Marciano, Harikumar Govindarajan, Amanda M. Williams, Brigitta Wastuwidyaningtyas, Thomas Bourquard, Panagiotis Katsonis, Olivier Lichtarge

**Affiliations:** a Department of Molecular and Human Genetics, Baylor College of Medicine, Houston, TX 77030, USA; b Quantitative and Computational Biosciences Graduate Program, Baylor College of Medicine, Houston, TX 77030, USA; c Cancer and Cell Biology Graduate Program, Baylor College of Medicine, Houston, TX 77030, USA; d MAbSilico, Nouzilly, Centre, France, EU; e Computational and Integrative Biomedical Research Center, Baylor College of Medicine, Houston, TX 77030, USA

**Keywords:** SARS-CoV-2, COVID-19, coronavirus, epitopes, sequence analysis

## Abstract

Since the first recognized case of COVID-19, more than 100 million people have been infected worldwide. Global efforts in drug and vaccine development to fight the disease have yielded vaccines and drug candidates to cure COVID-19. However, the spread of SARS-CoV-2 variants threatens the continued efficacy of these treatments. In order to address this, we interrogate the evolutionary history of the entire SARS-CoV-2 proteome to identify evolutionarily conserved functional sites that can inform the search for treatments with broader coverage across the coronavirus family. Combining this information with the mutations observed in the current COVID-19 outbreak, we systematically and comprehensively define evolutionarily stable sites that may provide useful drug and vaccine targets and which are less likely to be compromised by the emergence of new virus strains. Several experimentally-validated effective drugs interact with these proposed target sites. In addition, the same evolutionary information can prioritize cross reactive antigens that are useful in directing multi-epitope vaccine strategies to illicit broadly neutralizing immune responses to the betacoronavirus family. Although the results are focused on SARS-CoV-2, these approaches stem from evolutionary principles that are agnostic to the organism or infective agent.

## INTRODUCTION

COVID-19 is a worldwide affliction. Since first being reported in December 2019 in Wuhan, Hubei province, China, the World Health Organization (WHO) has tallied more than 2 million COVID-19 related deaths and over 100 million infections worldwide (as of February 8^th^, 2021) (Dong et al., 2020). Although timely public health interventions can successfully curtail incidence, the threat of subsequent waves of infections remains widespread and the emergence of new strains that evade current treatments is becoming a likely scenario (Dong et al., 2020; Kraemer et al., 2020; Wibmer et al., 2021; Wu et al., 2021; J. Zhang et al., 2020). The novel betacoronavirus (SARS-CoV-2) that is causing the pandemic is closely related to other known human coronavirus pathogens SARS-CoV, MERS-CoV (Chan et al., 2020; Lu et al., 2020), HCoV OC43, HKU1 and is more distantly related to the human infectious alphacoronaviruses HCoV 229E and HCoV NL63 (Lei et al., 2018). Finding ways to control and prevent further infection are top priorities which include the targeted discovery of drugs that impair viral mechanisms (Youngchang Kim et al., 2020; Li et al., 2020; Rut et al., 2020) and antigenic epitopes through which vaccines raise immunity (Mullard, 2020; Poh et al., 2020; van Doremalen et al., 2020). This study addresses both by utilizing evolutionary information from SARS-CoV-2 sequence and structural data to search for actionable functional sites for each protein in the SARS-CoV-2 genome.

In a first application, we note that the approval of new drugs under normal circumstances often takes more than 10 years (Dhama et al., 2020; Pillaiyar et al., 2020). In order to hasten the response, many current clinical trials for COVID-19 enlist antiviral agents that have targeted Zika, SARS-CoV, Ebola, and MERS-CoV in the past (Dhama et al., 2020; Jogalekar et al., 2020). In order to test more varieties of potential drugs, some studies screened thousands of clinical-stage or FDA-approved small molecules for antiviral activity, hoping to repurpose some of the top hits for COVID-19 treatment (Riva et al., 2020; White et al., 2021). However, the antiviral activity in these large-scale screens may, in part, be cell-line specific (Hoffmann et al., 2020), and therefore of unclear clinical relevance. Another approach to screen potential drugs for repurposing is to perform docking (Goodsell et al., 2020) of clinical-stage or FDA-approved drugs to the SARS-CoV-2 proteome (S. Gupta et al., 2020; Ortega et al., 2020). However, selection of the correct binding sites on the target proteins is crucial and difficult as protein surface cavities far exceed actual ligand binding sites that modulate function (Gupta et al., 2018). Here we systematically suggest potential drug target sites for most SARS-CoV-2 proteins based on evolutionary information. As these sites are chosen for their conserved functional roles, broad pan-coronavirus/betacoronavirus relevance, and minimal variability across all known SARS-CoV-2 variants, they should be prioritized in docking studies for drug repurposing.

In a second application, we note that understanding the immune response to SARS-CoV-2 infection is critical for vaccine development (Grifoni et al., 2020b). Most early SARS-CoV-2 immune epitope discovery studies rely heavily on bioinformatic prediction tools as well as sequence and epitope work already done in SARS-CoV and MERS-CoV. B-cell linear and discontinuous epitope prediction tools have been used by researchers to identify possible SARS-CoV-2 epitopes (Ahmed et al., 2020; Bhattacharya et al., 2020; Grifoni et al., 2020a). Several more recent studies experimentally determined SARS-CoV-2 immune epitopes (Le Bert et al., 2020; Nolan et al., 2020; Poh et al., 2020). Interestingly, several groups have reported significant T-cell reactivity against SARS-CoV-2 epitopes in individuals without virus exposure (Grifoni et al., 2020b; Le Bert et al., 2020; Mateus et al., 2020; Meckiff et al., 2020). Mateus et al. suggested that this could be due to cross reactivity between SARS-CoV-2 and other common human coronaviruses, such as OC43, HKU1, NL63 and 229E (Mateus et al., 2020). Here we report an evolutionary metric, which can accurately separate cross-reactive epitopes from those that are not, and use this metric to suggest potential cross-reactive epitopes in SARS-CoV-2. Prioritizing these cross-reactive epitopes in vaccine development can potentially lead to broadly neutralizing immunity across the betacoronavirus family, provide starting points for mAb development and offer guidance in updating current vaccines in the face of variants which make them less effective. The AstraZeneca, Moderna, and Pfizer vaccines currently being administered target the SARS-CoV-2 Spike glycoprotein (Baden et al., 2021; Polack et al., 2020; Voysey et al., 2021), while the cross-reactive epitopes highlighted here occur in less variable regions of the Spike protein and in other SARS-CoV-2 proteins.

Here, we use the Evolutionary Trace (ET) method, which predicts the importance of protein sequence positions, from most important (0.0) to least important (100.0). This relative phylogenetic ranking reflects the variation entropy of each sequence position within and across the branches of an associated evolutionary tree, revealing evolutionary pressure points that correspond to functional and structural determinants, and the protein sites at which they often cluster (Mihalek et al., 2004). Many studies validated this approach for predicting binding and catalytic functional sites (Lichtarge et al., 1996; Onrust et al., 1997), guiding protein engineering (Peterson et al., 2015; Shenoy et al., 2006; Sowa et al., 2001, 2000) and predicting function (Amin et al., 2013). When viewed as gradient of the evolutionary landscape, ET rankings of residue importance can be combined with amino acid substitution log odds to estimate the likely impact, or Evolutionary Action (EA), of coding variations on protein function (Katsonis and Lichtarge, 2019, 2017, 2014). Here, this first ET and EA analysis of a full viral proteome identifies evolutionary important residues and functional sites in SARS-CoV-2.

## RESULTS

### Evolutionary Trace of SARS-CoV-2.

**Figure 1.**
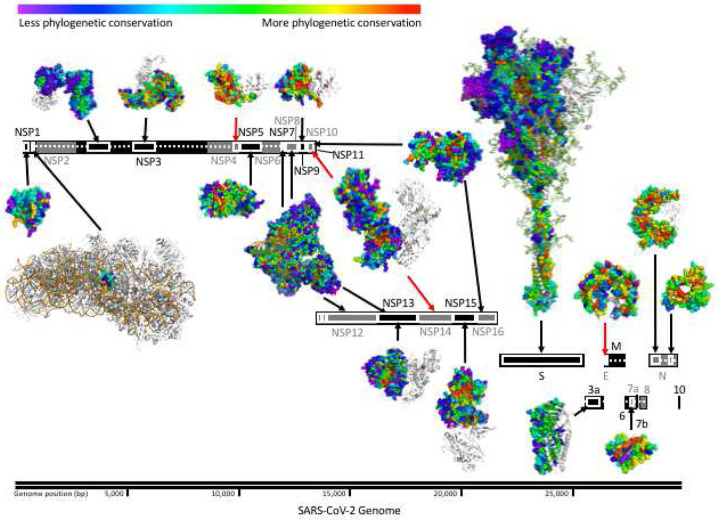
Structural and sequence information permits identification of evolutionarily important sites in SARS-CoV-2. Linear representation of SARS-CoV-2 proteome with structurally determined regions highlighted (white boxes) and corresponding structures’ residues colored by Evolutionary Trace rank. Black arrows connect SARS-CoV-2 structures to their corresponding gene, red arrows indicate that only structures of homologous proteins are available. Host ribosomal proteins in the NSP1 complex structure are shown in white. For multimeric structures, one monomer is also shown in white. Structures shown include: 7k7p (Clark et al., 2020) (NSP1), 6zlw (Thoms et al., 2020) (NSP1 C-term), 6woj (Alhammad et al., 2020) (NSP3), 6w9c (Osipiuk et al., 2020) (NSP3), ExPasy NSP4 model 01 (Bienert et al., 2017; “Non-structural protein 4 (nsp4) | P0DTD1 PRO_0000449622 | Models,” n.d.; Studer et al., 2020; Waterhouse et al., 2018) (NSP4), 6yb7 (Owen et al., 2020) (NSP5), 6wxd (Littler et al., 2020) (NSP9), 6xez (Chen et al., 2020) (NSP7, 8 , 12, and 13), 6zsl (Newman et al., 2020) (NSP13), 5c8s (Ma et al., 2015) (NSP10 and 14), 6wlc (Y. Kim et al., 2020) (NSP15), 6w4h (Minasov et al., 2020b) (NSP10 and NSP16), 6vsb_1_1_1 (Woo et al., 2020) (S), 6xdc (Kern et al., 2020) (ORF3a), 5x29 (Surya et al., 2018) (E), 6w37 (Nelson et al., 2020) (ORF7a), 6vyo (Chang et al., 2020) (N), and 6zco (Zinzula et al., 2020) (N).

In order to map functional sites and determinants in SARS-CoV-2 proteins we applied ET. With the multiple sequence alignments (Figure S1A, Dataset S1) and the corresponding phylogenetic trees (Figure S2-S4) in hand for 24 of the 26 SARS-CoV-2 proteins (see SI Methods and Materials), our protocol calculated the ET ranking of importance for 99.5% of SARS-CoV-2 amino acid residue positions (Dataset S2) generated from each of three protein databases (UniRef90, UniRef100, NCBI NR) and combined them into a single average. To independently assess the quality of these ranks, rather than rely on the variety and breadth of sequences in the alignments as indicative of information content, we used a statistical measure that quantifies the distribution of ET rankings in the 3D structure; the selection Cluster Weighting (SCW) z-score (Mihalek et al., 2004). This metric measures how well top-ranked ET residues cluster structurally relative to a randomized distribution of scores on the structure (see SI Materials and Methods). In previous studies, residues with better ET rankings (closer to 0) tended to cluster together at active sites, protein-protein interaction sites or other functional sites (Lichtarge et al., 1996; Mihalek et al., 2007, 2004; Wilkins et al., 2013, 2010). Here, such clustering of top-ranked residues was particularly prominent in several SARS-CoV-2 proteins and complexes including the NSP5 main protease, the NSP7/NSP8/NSP12 RNA-dependent RNA polymerase complex and the NSP10/NSP16 RNA cap methyltransferase complex and can be visualized as groups of warm colored residues in the protein structure (Figure 1). For almost all proteins, the SCW *z*-score is 2 standard deviations above the randomized background, confirming that the alignments are informative and that the resulting ET rankings are meaningful (Figure S1, Dataset S3). For the proteins that do not reach significant *z*-scores there is a clear correlation to a lack of sequences in the alignments (e.g. NSP1, E, ORF3, and ORF7a), or, the structure belongs to a small domain within a larger protein (e.g. the macrodomain within NSP3 and the HR2 domain within the S protein).

To probe these smaller domains within large proteins we further investigated the ADP-ribose-phosphatase (ADPRP) subdomain and macro and papain-like protease (PL^pro^) domains of NSP3. NSP3 was an intriguing case because top-ranked ET residues cluster well in its PL^pro^ domain but not in its macrodomain or in the ADPRP subdomain (Dataset S3). In order to better resolve ET rankings for NSP3, we generated new alignments, phylogenetic trees, and ET residue rankings for the subsequences specific to each NSP3 domain structure (see SI Materials and Methods). In this focused analysis, the PL^pro^ domain now yielded ~50% more sequences leading to a corresponding increase in the clustering of top-ranked residues (Figure S5). For the macrodomain and ADPRP subdomain, thousands of additional sequences spanning the three domains of life and distantly related viruses were included in the new data set which resulted in ET rankings that rivaled the significance of clustering in the PL^pro^ domain. The stark differences we find in the phylogenetic trees of specific NSP3 domains confirm previous observations of alternate domain configurations in different coronavirus genera and even within clades of betacoronavirus (Lei et al., 2018). The improvement in SCW *z*-score corresponds to a cluster of highly ranked ET residues within the ligand binding site of the macro domain and ADPRP subdomain (Figure S5D and E) which was missing in the analysis of the full NSP3 reference sequence. Having better resolved ET rankings in the NSP3 domains, we returned to the main data set to see how well ET rankings captured functional sites in other proteins.

### Phylogenetically conserved ligand binding sites.

**Figure 2.**
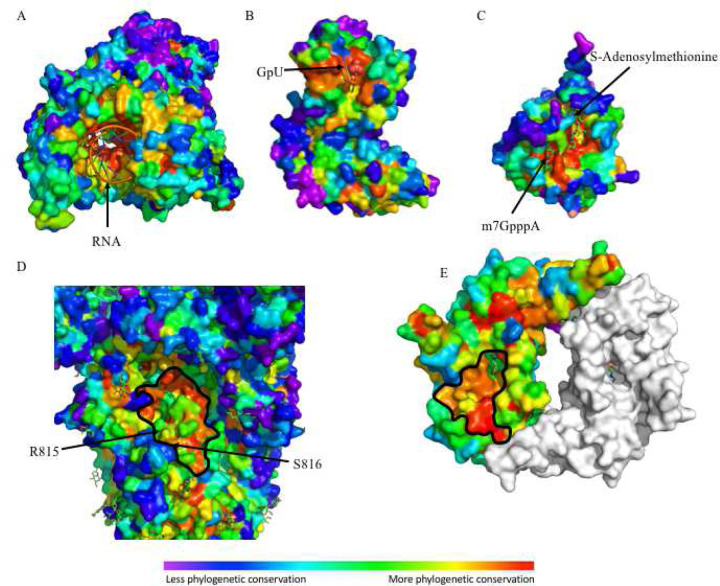
Top ET ranking residues overlap with known functional sites. ET recovers (A) the RNA binding site of NSP12 (RNA dependent RNA polymerase, pdb:6xez), (B) the active site of NSP15 (uridine-specific endoribonuclease, pdb:6x1b), (C) the substrates binding sites of NSP16 (RNA-cap methyltransferase, pdb:6wvn). ET also recovers a key functional (D) S2’ protease cleavage site of S (key residues: R815 and S816, pdb:6vsb), and predicts (E) a site associated with the putative RNA binding site of N (pdb:6vyo). The S cleavage site between R815 and S816 is labeled and the putative sites of panels D and E are highlighted with a black outline. The site in panel D is a high priority target as it was found that residues 815–825, which overlap this site, comprise the most frequently recognized epitope among naïve and COVID-19 patients (Shrock et al., 2020).

A catalog of SARS-CoV-2 ligand binding sites could serve as a timely resource for prioritizing therapeutic targets. Previous studies have shown that evolutionary sequence information correlates well-enough with enzyme active sites so as to serve as 3D-templates for functional signatures (Amin et al., 2013) and identify allosteric sites (Bhat et al., 2020; Rodriguez et al., 2010). Here we used NSP12, NSP15 and NSP16 as examples to show how the evolutionary sequence information captured by ET can successfully predict ligand binding sites for virus proteins. NSP12 is an RNA dependent polymerase, NSP15 mediates the cleavage of both single- and double-stranded RNA at uridine sites (Ulferts and Ziebuhr, 2011) and NSP16 is a m7GpppA-specific, S-adenosylmethionine (SAM)-dependent, 2’-O-MTase (Decroly et al., 2011). As shown in Figure 2A–C, top ranked ET residues cluster around the native ligands of NSP12 (RNA) (Liu et al., 2020), NSP15 (GpU) (Youngchang Kim et al., 2020) and NSP16 (m7GpppA and SAM) (Rosas-Lemus et al., 2020), indicating an accurate prediction of ligand binding sites for these proteins. In order to quantify this result, the enrichment of highly ranked ET residues (ET ranking <= 30) around the ligand binding sites (within 5Å of the ligand) of NSP3, NSP12, NSP13, NSP14, NSP15 and NSP16 was ascertained by a Fisher’s exact test. In each case, there is a statistically significant enrichment (Figure S6 and Dataset S4). Using this statistical test, we also find that in nine of the eleven ligand binding sites analyzed, our analysis better identifies known ligand binding sites than a previously reported analysis of the SARS-CoV-2 proteome using a percent identity metric (Figure S6 and Table S4) (R. Gupta et al., 2020). Moreover, several new functional sites are also predicted by ET (Figure 2D and 2E). On the spike protein (S), one such ET cluster partially overlaps the S2’ protease cleavage site and fusion peptide that is critical for membrane fusion and infectivity of the SARS virus (Madu et al., 2009). On the N-terminal domain of the nucleoprotein (N), a cluster of highly ranked ET residues are either adjacent to (Dinesh et al., 2020) or overlap (Lin et al., 2014; Saikatendu et al., 2007) the putative RNA binding site and may contribute to formation of the N protein-RNA helical filaments that package the RNA genome (Chen et al., 2007). These results indicate ET can provide alternative drug target sites with no currently available ligand-bound structures.

**Figure 3.**
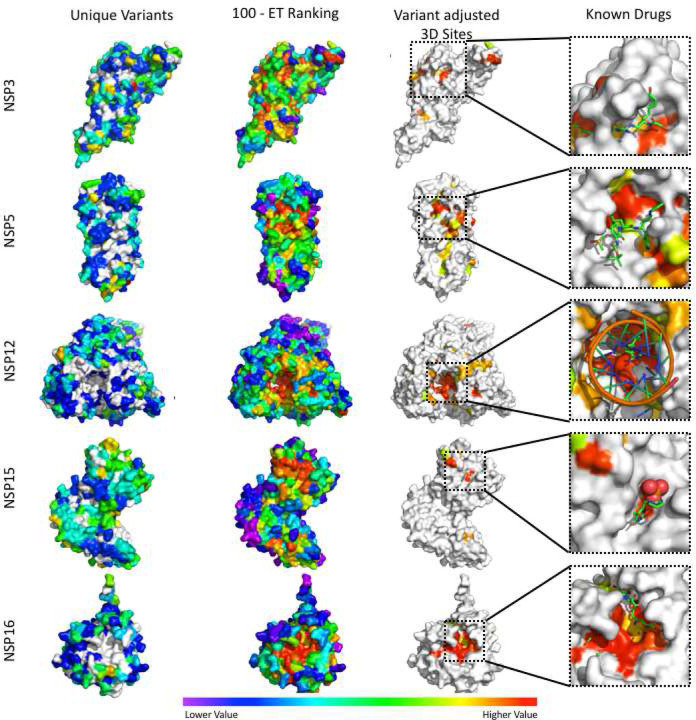
Identification of variant adjusted 3D sites (5Å) and their colocalization with known drug binding sites. Variant adjusted 3D sites for NSP3 (6w9c(Osipiuk et al., 2020)), NSP5 (6yb7(Owen et al., 2020)), NSP12 (7bv1(Yin et al., 2020)), NSP15 (6wlc(Youngchang Kim et al., 2020)), and NSP16 (6w4h(Minasov et al., 2020b)) were identified as clusters of surface residues with low ET ranks and a lack of mutations in the current outbreak. In the known drugs panels, variant adjusted 3D sites were identified using apo form structures, then mapped to the co-structures of NSP3 with peptide inhibitor vir251 (PDB:6wx4(Rut et al., 2020)), NSP5 with potential drug 13b (PDB:6y2f(L. Zhang et al., 2020)), NSP12 with drug remdesivir (7bv2(Yin et al., 2020)), NSP15 in complex with potential drug tipiracil (PDB:6wxc(Youngchang Kim et al., 2020)), and NSP16 with sinefungin (PDB:6wkq(Minasov et al., 2020a)). For structures in the “Unique Variants” column “Lower Values” in the color scale correspond to fewer variants, while “Higher Values” correspond to more variants and white residues have no reported variants in the analyzed SARS-CoV-2 strains. For the “100 – ET Ranking”, “Variant Adjusted 3D Sites”, and “Known Drugs” columns, “Lower Values” correspond to less phylogenetic conservation while “Higher Values” correspond to more phylogenetic conservation.

In addition to being important to protein function, ideal drug target sites should also be rarely mutated in the current outbreak so as to avoid the potential emergence of drug resistance. Thus, we focused on positions that do not have any mutations observed in the 139,607 high quality, full length SARS-CoV-2 sequences that were available as of December 8th, 2020. As more genomes and mutations within them are sequenced it may be necessary to lower the variant count stringency. In order to translate proteome-wide ET ranks and mutational profiles into useful information for drug development, we defined clusters of mutation-free, surface-exposed residues that are highly ranked by ET and fall within 5Å of each other (Figure 3, Dataset S5) as variant adjusted 3D sites. The resulting catalog of potential drug targets includes 103 sites at ~4 sites per structure with the largest structure (full-length model of Spike, 6vsb_1_1_1) having the highest number of sites. For NSP12, NSP15 and NSP16, the variant adjusted 3D sites overlap the known ligand binding sites.

In order to evaluate whether these variant adjusted 3D sites may correspond to druggable target sites, we examined their overlap with sites observed in five SARS-CoV-2 protein-drug complex crystal structures. It is important to note that all 5 drugs showed an inhibitory effect in either cellular or biochemical assays. Remdesivir has been shown to speed up the recovery of COVID-19 patients in clinical trials (Beigel et al., 2020), while the α-ketoamide inhibitor 13b can suppress SARS-CoV-2 replication in cell lines (L. Zhang et al., 2020). Vir251 and tipiracil were also shown to effectively inhibit the enzymatic activities of their targets (Youngchang Kim et al., 2020; Rut et al., 2020). The remaining drug, sinefungin, is a pan-MTnase (NSP16) inhibitor that inhibits the growth of yeast cells ectopically expressing NSP16 from SARS-CoV (Decroly et al., 2011). The variant adjusted 3D sites were mapped onto the five SARS-CoV-2 protein-drug complexes (Youngchang Kim et al., 2020; Minasov et al., 2020a; Rut et al., 2020; Yin et al., 2020; L. Zhang et al., 2020) and, as shown in Figure 3, all five drugs reside in protein surface pockets that are within or are very close to at least one residue of a predicted variant adjusted 3D site. The variant adjusted 3D site for NSP5 is not well recovered mostly due to a single SARS-CoV-2 sequencing entry (strain MT745875) wherein several residues in the protease active site are mutated (G143S, S144E and C145I), including the catalytic cystine residue. S144E and C145I are both caused by two nucleotide substitutions in the codon, and only observed in this strain (sampled on 06/24/20). It is unclear whether this is a sequencing artifact or represents a genuine active site plasticity that compromises NSP5’s active site as a stable drug target. It does however illustrate the importance of accurately detecting emerging sequence variations when choosing drug targets. A clearer example of this is the tipiracil bound NSP15 active site which, although evolutionarily important to the overall coronavirus family, is not predicted to be a good drug target due to the presence of multiple variants observed in the current outbreak. Overall, these results show that predicted variant adjusted 3D sites can recover experimentally tested drug binding pockets and suggest new sites that can be targeted in computational docking approaches. In addition, because these sites are conserved across multiple coronavirus genera, these predicted variant adjusted 3D sites are anticipated to be relevant for identifying inhibitors of SARS-CoV-2 as well as more distantly related coronaviruses.

### Conserved linear sites.

**Figure 4.**
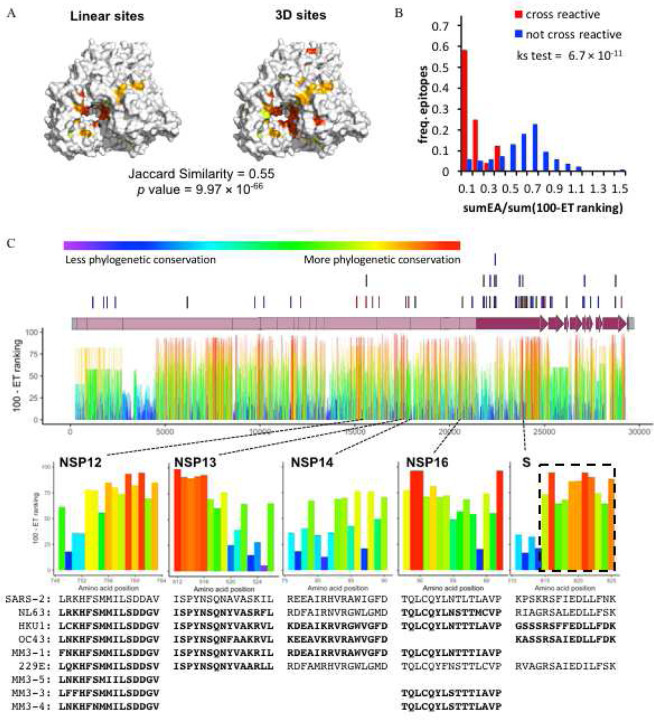
Identification of variant adjusted linear sites with Evolutionary Trace. A) Mapping of variant adjusted linear and structural sites on the surface of NSP12 (7bv1) with Jaccard Similarity value and Fisher’s Exact test *p* value indicated. B) Relative frequency distributions of sumEA/sum(100-ET ranking) for T-cell epitopes shown to either be cross reactive (red) or not (blue). The sumEA/sum(100-ET ranking) metric predicts the functional impact of variants (EA) relative to the overall Evolutionary Trace rankings in the epitope. A Kolmogorov-Smirnov test (ks test) shows a significant difference in the distributions. C) T-cell epitopes reported in Mateus, et al. (Mateus et al., 2020) are shown above the SARS-CoV-2 genome (lines) and ET rankings within each protein are shown below. Shown are five SARS-CoV-2 epitopes (NSP12, NSP13, NSP14, NSP16 or S) that are predicted to cross react with the indicated common human coronavirus epitopes (bold text). Closely related coronavirus epitopes that did not meet our stringent threshold are also shown (normal text). The dashed box highlights the 11 amino acid stretch subsequently shown to be the most cross-reactive Spike protein epitope among naïve and COVID-19 patients (Shrock et al., 2020).

Variant adjusted 3D sites may prove valuable in guiding drug design, but these approaches are dependent upon having high resolution crystal structures and some structures are either not yet available (e.g. NSP2, NSP6, M, and several accessory proteins), do not cover a majority of the protein (NSP3 and NSP4) or are too low in resolution for accurate docking studies (NSP12, NSP14, ectodomain of S, N, ORF3a and ORF7a). However, ET operates over protein sequences and can therefore identify phylogenetically important linear sequence fragments even in the absence of a 3D structure (Lichtarge et al., 2002). As in our approach to discover variant adjusted 3D sites, we combined ET residue ranking information with sequencing data from SARS-CoV-2 isolates to arrive at linear peptides along the proteome that are evolutionarily important and also show no variation in the current outbreak (Figure S7, Dataset S6). In order to assess the value of these variant adjusted linear sites, we asked whether they could recapitulate variant adjusted 3D sites. Variant adjusted linear sites for NSP12 were mapped onto an available NSP12 structure and, as illustrated in Figure 4A, the majority of the 3D and linear sites overlap with each other. Variant adjusted linear sites and 3D sites overlap well for other SARS-CoV-2 proteins, which was quantified by Jaccard Similarity and Fisher’s exact test (Dataset S7). These data suggest that variant adjusted linear sites contain functionally relevant information since they recapitulate variant adjusted 3D sites for proteins or domains without requiring 3D structural data. In the absence of a protein structure, these linear sites could be useful in designing inhibitory peptides (Gu et al., 2005; Wu et al., 2020).

These peptides are also connected to a second main approach towards resolving the pandemic, vaccine and mAb development. Although vaccines for COVID-19 are now available, several new variants that arose in the UK (B.1.1.7) (Rambaut et al., 2020) and South Africa (B.1.351, also known as 501Y.V2) (Tegally et al., 2020) have multiple substitutions in the Spike protein’s receptor binding domain. Both are resistant to several classes of mAbs (Ho et al., 2021; Wibmer et al., 2021). While B.1.1.7 is ~ 2 fold more resistant to convalescent plasma, B.1.351 is more concerning as it can be ~11–33 fold more resistant to the convalescent plasma obtained from ~80% of patients (Ho et al., 2021; Wibmer et al., 2021). Ideally, effective protection against future outbreaks from related coronaviruses would include a broadly neutralizing effect wherein the immune system recognizes epitopes shared among coronavirus species. The prospect of raising a broad antibody response is bolstered by a study that naïve patients, never exposed to SARS-CoV-2, were found to possess a subset of T-cells that can cross-react to homologous epitopes shared by common cold coronaviruses and SARS-CoV-2 (Mateus et al., 2020). In this context, we note that ET rankings reflect the degree of homology over the phylogenetic tree, so we reasoned that summing ET scores over the length of an identified T-cell epitope may be able to estimate its potential for cross-reactivity.

As a first step, we summed the ET ranks for each of the 40 SARS-CoV-2 epitopes that had been shown to react with patient-derived T-cells so that they could be ranked by predicted cross-reactivity to 161 common cold coronavirus epitopes assayed by Mateus et al. Although summing ET ranks could identify SARS-CoV-2 epitopes that are more likely to be cross-reactive (Figure S8), it did not account for the specific amino acid differences in the potentially cross-reactive homolog. In other words, ET ranks can predict whether or not a SARS-CoV-2 epitope will be cross-reactive in general, but not which epitope homologs will cross react.

In order to improve resolution of our predictions to specific epitope homologs, we next combined EA, a predictor of mutational impact, with the summed ET rankings. EA calculates the predicted impact of amino acid variations on protein function aiding in the interpretation of coding variants (Katsonis and Lichtarge, 2019, 2017, 2014). Summing the predicted impact of amino acid changes between a SARS-CoV-2 epitope and a homologous epitope in another virus (sumEA) while adjusting for the SARS-CoV-2 epitope’s overall evolutionary importance (sum(100-ET ranking)) produced a metric that was able to separate cross-reactive epitopes from those that did not cross react (Figure 4B and S8, Dataset S8). This metric, sumEA/sum(100-ET ranking), was then applied to 21 untested SARS-CoV-2 T-cell epitopes and their common cold homologs (Mateus et al., 2020). From a total of 92 homologs we identified 23 with potential to cross react to one of five SARS-CoV-2 epitopes (Figure 4C, Dataset S9). These 5 SARS-CoV-2 epitopes along with the 9 others experimentally shown to possess cross-reactivity could be used in a multi-epitope vaccination strategy that provides a broad neutralizing response to currently circulating coronaviruses, SARS-CoV-2 and, possibly, future outbreaks. Subsequent to this analysis, further confirmation came when it was found that residues 815–825 of the Spike protein compose the most frequently recognized epitope among naïve and COVID-19 patients (Shrock et al., 2020). These 11 residues are specifically highlighted by ET as being particularly evolutionarily conserved amino acids and are thereby responsible for our metric’s prediction of cross reactivity in the 15 amino acid long epitopes used in the study by Mateus et al. This result and the generality of our approach suggest highly cross-reactive epitopes could be quickly identified in other families of pathogens.

### Dissemination.

To disseminate these results, a public website (http://cov.lichtargelab.org) makes these data and analyses fully accessible. The data include, for example, multiple sequence alignments, pre-calculated ET ranks, and predicted sites (both linear and structural) for all SARS-CoV-2 proteins. In addition, an interactive structure viewer enables users to explore ET-colored structures and predicted variant adjusted ET sites associated with those structures (Dataset S5-6). The website will be updated as new SARS-CoV-2 isolates and protein structures become available.

## DISCUSSION

Rapid progress has been made in response to the SARS-CoV-2 pandemic; from sequencing, to structural determination, and drug and vaccine development (Jeong et al., 2020; Kneller et al., 2020; Li et al., 2020; Sanders et al., 2020; Wu et al., 2020). Here, we make use of coronavirus phylogenetics, and sequence and structure information to provide a functional map of sites that are not only stable across coronavirus families but are also stable to mutations in the current pandemic. These sites are favored strategic targets for pan coronavirus/betacoronavirus therapeutics that are less likely to be subjected to the rapid emergence of resistance from SARS-CoV-2 variants. In addition to focusing therapeutic studies, the data presented here can guide directed mutagenesis studies aimed at identifying the mechanism of action for successful therapies, not only in the context of the current outbreak but across future coronavirus outbreaks.

There are limitations to this study. The quality of our results depends on the number and range of homologous sequences available and a few of the SARS-CoV-2 proteins such as NSP1 and the accessory proteins do not reach significant z-scores or have many diverse sequences in their final alignments. The inability to recover more sequence information could be due to a higher evolutionary rate in these proteins that limits our ability to recognize distantly related homologs with very little sequence identity. More likely, these peripheral genes have been more recently recruited through the frequent recombination events that occur in the coronavirus family (Su et al., 2016). The equivalent of gene recruitment has occurred at the domain level in the NSP3 protein with its variable number of domains (10 to 16), some of which are unique to the betacoronavirus clade b containing SARS-CoV and SARS-CoV-2. Therefore, it is unsurprising that the initial alignments and corresponding ET rankings for full-length NSP3 are heavily influenced by the less divergent PL^pro^ domain that is present across coronavirus clades and families. Domain-specific analysis of NSP3 greatly improved both the number of sequences returned, phylogenetic coverage, and the resolution of ET results. This suggests that future work should include domain specific analyses for multidomain proteins. Such analyses are likely to provide ET rankings that identify important functional sites for individual domains, while full-length analyses can provide insight into how particular domains were recruited in specific branches of the phylogenetic tree.

Several other groups have focused on experimentally screening clinical-stage or FDA-approved small molecules with the hope of identifying and repurposing drugs for SARS-CoV-2 treatment. However, drug efficacy of top hits might be cell line specific (Hoffmann et al., 2020) and the mechanisms of drug action may be unclear or acting through modulation of the tissue culture cell. In silico docking studies (Deshpande et al., 2020; S. Gupta et al., 2020) take a more targeted approach towards specific SARS-CoV-2 sites and benefit from knowledge of ligand binding sites. Although structural characterization of SARS-CoV-2 proteins is unprecedented, the structural information available is far from comprehensive. In order to bridge these knowledge gaps, we identified 3D clusters of surface residues that have low ET rankings and a lack of mutations in the current outbreak as potential drug target sites. Many of these variant adjusted 3D sites correspond to ligand bound active sites, but others map to evolutionarily important sites that have yet to be fully characterized. These variant adjusted sites are putative drug targets which can guide docking studies to sites not immediately apparent from currently available structural information.

The depth of the phylogenetic tree for the sequences analyzed with ET can set expectations for how broadly a drug may inhibit homologs in different species. For instance, the active site of NSP12 is conserved throughout a deep phylogenetic tree of RNA viruses and an inhibitor targeting it, remdesivir, is effective against SARS-CoV-2, SARS-CoV, MERS and the distantly related Ebola RNA virus (de Wit et al., 2020; Eastman et al., 2020). Likewise, most of the other NSPs and the structural M (membrane) and N (nucleocapsid) proteins have deep RNA virus phylogenies and targeting them may provide broadly effective inhibitors. The most obvious candidates are the variant adjusted 3D sites that overlap with the ligand binding sites of NSP12, NSP13, NSP14 and 16. A less apparent drug target revealed by our analysis includes a putative RNA binding site on the N terminal domain of the N protein (Kang et al., 2020). The equivalent site in HCoV-OC43’s N protein is targeted by compound PJ34 where it inhibits RNA binding activity and viral replication (Lin et al., 2014; Peng et al., 2020). In contrast, another inhibitor (5-benzyloxygramine), that induces aggregation of the MERS-CoV N protein and shows potent antiviral activity against that virus (Lin et al., 2020), binds two hydrophobic pockets. However, these pockets have undergone variation in the current SARS-CoV-2 outbreak, suggesting compounds targeting these areas are more likely to become susceptible to resistant SARS-CoV-2 variants.

In contrast to the aforementioned deep phylogenies predominantly composed of RNA virus sequences, the ADP ribose phosphatase sub-domain of NSP3 has a phylogenetic tree with few coronavirus sequences among a multitude of sequences that span three domains of life. Drugs targeting this domain may inhibit coronavirus infectivity but could also inhibit host ADP ribose phosphatases. ADP ribose phosphatase inhibitors have already been developed for cancer treatment and their application towards SARS-CoV-2 treatment is warranted (Kassab et al., 2020) but care should be taken to ensure unwanted side effects do not overshadow any benefits as a viral inhibitor.

Along with small molecule viral inhibitors, the development of immunological therapeutics to address COVID-19 can also be guided by the use of evolutionary information. We performed evolutionary analysis on SARS-CoV-2 T-cell epitopes capable of cross reacting with homologous peptides in other human coronaviruses (Le Bert et al., 2020; Mateus et al., 2020). This led to a new metric, sumEA/sum(100-ET ranking), that can better predict which epitopes will cross-react. In general, knowledge of cross-reactive epitopes could inform multi-epitope vaccine development efforts to direct the immune system towards a broadly neutralizing response.

The S protein evolves to bind different receptors (Fehr and Perlman, 2015), suggesting the high variation rate of the binding site is due to both this adaptive function and the avoidance of the host’s adaptive immune system. Though several vaccines and mAbs are now in use, new strains are arising rapidly (Faria et al., 2021; Fiorentini et al., 2021; Rambaut et al., 2020; Tegally et al., 2020) and show signs of evading existing treatments (Wibmer et al., 2021; Wu et al., 2021). The two most worrisome mutations in those strains occur at N501 and E484 in the receptor binding domain of the S protein. These residues have ET ranks of 96 and 95.8 respectively, indicative of particularly rapid phylogenetic change across the coronavirus family and represent poor targets for therapeutics meant to remain effective against emerging SARS-CoV-2 variants. Despite the variability of the S protein, we identified a relatively conserved site (Figure 2D) that corresponds to a fusion peptide adjacent to the S2’ cleavage site (Madu et al., 2009) that is also the most cross-reactive epitope among naïve and COVID-19 patients (Shrock et al., 2020) (Figure 4C). This site is particularly appealing when considering that the 5H10 human mAb targeting the equivalent region in SARS-CoV was very effective in preventing disease a *Rhesus macaque* infection model (Miyoshi-Akiyama et al., 2011). The appearance of new strains makes it very likely that additional vaccine and mAb development will be necessary. We believe that targeting the SARS-CoV-2 variant-adjusted linear ET site corresponding to the S2’ cleavage site and fusion peptide region and other sites highlighted by our study, may provide protection that is less susceptible to the emergence of resistant variants.

## CONCLUSION

This study was motivated by the current pandemic and uses evolutionary sequence information to guide the development of therapeutics for COVID-19. Although we are presently in the grip of COVID-19, this pandemic was preceded by the SARS and MERS outbreaks and it should be anticipated that related coronaviruses will cause future outbreaks. And while this study is focused upon SARS-CoV-2, it draws upon pieces of sequence information taken from the whole of the coronavirus family and thereby the findings are extendable to other coronavirus species, including those that have not yet been encountered. Indeed, the tools we present could be applied to any family of pathogen. Putting a pandemic virus into the evolutionary context of related viruses can expose a path to managing recovery and may offer therapeutics that cover future outbreaks.

## MATERIALS AND METHODS

A brief description of the methods can be found here; for a more in-depth description of specific methods please see the Supplementary text.

### Evolutionary Trace:

In order to map functional determinants in SARS-CoV-2 proteins we applied the Evolutionary Trace (ET) approach (Lichtarge et al., 1996; Mihalek et al., 2004). This method ranks each amino acid position from most to least important during evolution by tracking how they vary along the coronavirus phylogenetic tree. These rankings vary based on the precise choice of multiple sequence alignment (MSA). In order to produce robust ET rankings three separate alignments were generated for each protein in the SARS-CoV-2 Wuhan-Hu-1 reference genome (NC_045512.2) (Wu et al., 2020), by querying three protein databases (UniRef90, UniRef100, and NCBI NR) for sequences with identity between 25% and 98%. This procedure filtered out sequences that were either overly distant or redundant. Only two proteins had too few matches for ET, NSP11 and ORF10, both of which have unknown function (Gadhave et al., 2021; Pancer et al., 2020) and have very short reference sequences (13 and 38 amino acids, respectively, FigureS1, Dataset S1). The ET scores for all other proteins for each alignment and for the average scores across alignments were evaluated with the previously presented Selection Cluster Weighting (SCW) z-score (Mihalek et al., 2007, 2004; Wilkins et al., 2013, 2010). The *z*-scores for each structure were then ranked 1–4 in order to determine if ET scores from one database or the average of the three consistently outperforms the others. ET scores from each of the three databases performed similarly well but the average ET of the three provided better *z*-scores in most cases (Figure S1C). ET rankings were further investigated by comparing the highest scoring regions with known functional sites.

### Prediction of Variant Adjusted ET Sites:

Variant adjusted ET sites were predicted based on both the linear sequence as well as structural constraints. Residues were nominated as members of potential therapeutic sites based on their ET rankings, lack of variants as found in SARS-CoV-2 sequences retrieved from GISAID (Shu and McCauley, 2017), Genbank (Benson et al., 2018), and the China National Center for Bioinformation (CNCB) (Zhao et al., 2020), as well as surface accessibility, and structural proximity. Structurally identified therapeutic sites were compared to drug binding sites for agents known to bind to SARS-CoV-2 proteins. To generalize this approach to proteins without structure, linear sites were predicted based on ET rankings, current mutational profile and linear connectivity. Structural and linear predicted sites were compared to one another using Jaccard Similarity and Fisher’s Exact test, to determine the usefulness of this method in the absence of a protein structure. Several ET metrics were also interrogated to determine their ability to highlight potential cross-reactive immunogenic epitopes (Mateus et al., 2020). The best metric, sumEA/sum(100-ET ranking), was used to predict cross-reactive T-cell epitopes which are good potential therapeutic sites.

## Supplementary Material

Supplement 1 Other supplementary materials for this manuscript include the following:
Supplementary text with Supporting materials and methods, Figures S1 to S8 and Legends for Datasets S1 to S9
Datasets S1 to S9

## Significance Statement

By examining past evolutionary pressures in the coronavirus family and in the present SARS-CoV-2 outbreak, we identified functional sites in the SARS-CoV-2 proteome that are not affected by variants in the current outbreak. These sites can be targeted for small molecule docking, used for pan-coronavirus/betacoronavirus vaccine and monoclonal antibody (mAb) development, provide templates for mimetic peptides, and offer genetic targets to generate attenuated virus.
