# Supplementary material for: Identification of evolutionarily stable functional and immunogenic sites across the SARS-CoV-2 proteome and the greater coronavirus family
Source: Res Sq. 2021 Feb 15:rs.3.rs-95030. Originally published 2020 Oct 20. Preprint. [Version 3] doi: 10.21203/rs.3.rs-95030/v3 (PMC7587783; doi:10.21203/rs.3.rs-95030/v3)
Supplement: Supplement 1 — Other supplementary materials for this manuscript include the following: Supplementary text with Supporting materials and methods, Figures S1 to S8 and Legends for Datasets S1 to S9 Datasets S1 to S9 [file NIHPPRS95030v3-supplement-1.pdf]

Supplementary Files

This is a list of supplementary files associated with this preprint. Click to download.

- ETCoV2R2SIv4.pdf
